# Supplementary material for: Safety and Immunogenicity of Inactivated Whole Virion COVID-19 Vaccine CoviVac in Clinical Trials in 18–60 and 60+ Age Cohorts
Source: Viruses. 2023 Aug 29;15(9):1828. doi: 10.3390/v15091828 (PMC10537914; doi:10.3390/v15091828)
Supplement: Supplementary file 1 [file viruses-15-01828-s001.zip › viruses-2480699-supplementary.pdf]

SUPPLEMENTARY MATERIALS

Supplementary Table S1. Adverse events severity score

Score	Hyperemia/swelling/induration at the injection site	Fever	Other
0 – absent	none	≤ 37.0 °C	none
1 – mild	hyperemia <50 mm or swelling/induration <25 mm	> 37.0 °C – ≤ 37.5 °C	does not interfere with daily activities
2 – moderate	hyperemia >50 mm or swelling/induration 26–50 mm	> 37.6 °C – ≤ 38.5 °C	interferes with daily activities
3 – severe	swelling/induration >50 mm	> 38.6 °C	prevents daily activities

Supplementary Table S2. Total adverse events observed in the Vaccine Group and in the Placebo Group within 28 days following each vaccination

Adverse event	Vaccine<br>Number of participants with AEs (%, 95% CI) / number of AEs			Placebo<br>Number of participants with AEs (%, 95% CI) / number of AEs		
	Total participants<br>N=298	Within 28 days after		Total participants<br>N=100	Within 28 days after	
		First dose	Second dose		First dose	Second dose
**Local and systemic reactions**	58 (19.5%, 15.4–24.3%) / 84	34 (11.4%, 8.3–15.5%) / 39	34 (11.4%, 8.3–15.5%) / 45	16 (16%, 10.1–24.4%) / 23	7 (7%, 3.4–13.7%) / 8	10 (10%) / 15
Pain at the injection site	48 (16.1%, 12.4–20.7%) / 56	31 (10.4%, 7.4–14.4%) / 31	25 (8.4%, 5.7–12.1%) / 25	11 (11%, 6.3–18.6%) / 12	5 (5%, 2.2–11.2%) / 5	7 (7%, 3.4–13.7%) / 7
Induration at the injection site	3 (1%, 0.3–2.9%) / 3	1 (0.3%, 0.1–1.9%) / 1	2 (0.7%, 0.2–2.4%) / 2	3 (3%, 1.0–8.5%) / 3	–	3 (3%, 1.0–8.5%) / 3
Hematoma at the injection site	–	–	–	1 (1%, 0.2–5.4%) / 1	1 (1%, 0.2–5.4%) / 1	–
Swelling at the injection site				1 (1%, 0.2–5.4%) / 1		1 (1%, 0.2–5.4%) / 1
Itching at the injection site				1 (1%, 0.2–5.4%) / 1		1 (1%, 0.2–5.4%) / 1
Fever	13 (4.4%, 2.7–7.3%) / 14	6 (2%, 0.9–4.3%) / 6	7 (2.3%, 1.1–4.8%) / 8	2 (2%, 0.6–7.0%) / 2	2 (2%, 0.6–7.0%) / 2	–
Malaise	5 (1.7%, 0.7–3.9%) / 11	1 (0.3%, 0.1–1.9%) / 1	4 (1.3%, 0.5–3.4%) / 10	2 (2%, 0.6–7.0%) / 3	–	2 (2%, 0.6–7.0%) / 3
**Laboratory methods**	22 (7.4%, 4.9–10.9%) / 25	8 (2.7%, 1.4–5.2%) / 8	14 (4.7%, 2.8–7.7%) / 17	7 (7%, 3.4–13.7%) / 7	2 (2%, 0.6–7.0%) / 2	5 (5%, 2.2–11.2%) / 5
Increased leukocytes content	1 (0.3%, 0.1–1.9%) / 1	1 (0.3%, 0.1–1.9%) / 1	–	–	–	–
Positive SARS-CoV-2 PCR test	18 (6%, 3.9–9.3%) / 18	7 (2.3%, 1.1–4.8%) / 7	11 (3.7%, 2.1–6.5%) / 11	6 (6%, 2.8–12.5%) / 6	2 (2%, 0.6–7.0%) / 2	4 (4%, 1.6–9.8%) / 4
Increased creatine phosphokinase level	3 (1%, 0.3–2.9%) / 3	–	3 (1%, 0.3–2.9%) / 3	1 (1%, 0.2–5.4%) / 1	–	1 (1%, 0.2–5.4%) / 1
Increased ALT level	1 (0.3%, 0.1–1.9%) / 1	–	1 (0.3%, 0.1–1.9%) / 1	–	–	–
Increased AST level	1 (0.3%, 0.1–1.9%) / 1	–	1 (0.3%, 0.1–1.9%) / 1	–	–	–
Increased CRP level	1 (0.3%, 0.1–1.9%) / 1	–	1 (0.3%, 0.1–1.9%) / 1	–	–	–
**Blood and lymphatic system disorders**	1 (0.3%, 0.1–1.9%) / 1	1 (0.3%, 0.1–1.9%) / 1	–			
Inguinal lymphadenitis	1 (0.3%, 0.1–1.9%) / 1	1 (0.3%, 0.1–1.9%) / 1	–	–	–	–
**Blood vessel disorders**				1 (1%, 0.2–5.4%) / 1	1 (1%, 0.2–5.4%) / 1	
Deep venous leg thrombosis	–	–	–	1 (1%, 0.2–5.4%) / 1	1 (1%, 0.2–5.4%) / 1	–
**Infections and parasitic invasions**	29 (9.7%, 6.9–13.6%) / 29	18 (6%, 3.9–9.3%) / 18	11 (3.7%, 2.1–6.5%) / 11	4 (4%, 1.6–9.8%) / 4	2 (2%, 0.6–7.0%) / 2	2 (2%, 0.6–7.0%) / 2
SARS-CoV-2 infection	25 (8.4%, 5.7–12.1%) / 25	16 (5.4%, 3.3–8.5%) / 16	9 (3%, 1.6–5.6%) / 9	4 (4%, 1.6–9.8%) / 4	2 (2%, 0.6–7.0%) / 2	2 (2%, 0.6–7.0%) / 2
Other upper respiratory tract infections	3 (1%, 0.3–2.9%) / 3	2 (0.7%, 0.2–2.4%) / 2	1 (0.3%, 0.1–1.9%) / 1	–	–	–
Candidiasis	1 (0.3%, 0.1–1.9%) / 1	–	1 (0.3%, 0.1–1.9%) / 1	–	–	–
**Nervous system disorders**	11 (3.7%, 2.1–6.5%) / 20	4 (1.3%, 0.5–3.4%) / 4	9 (3%, 1.6–5.6%) / 16	6 (6%, 2.8–12.5%) / 12	3 (3%, 1.0–8.5%) / 3	3 (3%, 1.0–8.5%) / 9
Headache	11 (3.7%, 2.1–6.5%) / 19	4 (1.3%, 0.5–3.4%) / 4	8 (2.7%, 1.4–5.2%) / 15	6 (6%, 2.8–12.5%) / 12	3 (3%, 1.0–8.5%) / 3	3 (3%, 1.0–8.5%) / 9
Dizziness	1 (0.3%, 0.1–1.9%) / 1	–	1 (0.3%, 0.1–1.9%) / 1	–	–	–
**Musculoskeletal and connective tissue disorders**	5 (1.7%, 0.7–3.9%) / 8	2 (0.7%, 0.2–2.4%) / 3	3 (1%, 0.3–2.9%) / 5	1 (1%, 0.2–5.4%) / 1	1 (1%, 0.2–5.4%) / 1	–
Arthralgia	3 (1%, 0.3–2.9%) / 3	1 (0.3%, 0.1–1.9%) / 1	2 (0.7%, 0.2–2.4%) / 2	–	–	–
Lumbar pain	–	–	–	1 (1%, 0.2–5.4%) / 1	1 (1%, 0.2–5.4%) / 1	–
Myalgia	5 (1.7%, 0.7–3.9%) / 5	2 (0.7%, 0.2–2.4%) / 2	3 (1%, 0.3–2.9%) / 3	–	–	–
**Gastrointestinal tract disorders**	3 (1%, 0.3–2.9%) / 4	1 (0.3%, 0.1–1.9%) / 1	2 (0.7%, 0.2–2.4%) / 3	1 (1%, 0.2–5.4%) / 1	–	1 (1%, 0.2–5.4%) / 1
Diarrhea	1 (0.3%, 0.1–1.9%) / 1	–	1 (0.3%, 0.1–1.9%) / 1	1 (1%, 0.2–5.4%) / 1	–	1 (1%, 0.2–5.4%) / 1
Pyrosis	1 (0.3%, 0.1–1.9%) / 1	1 (0.3%, 0.1–1.9%) / 1	–	–	–	–
Nausea	2 (0.7%, 0.2–2.4%) / 2	–	2 (0.7%, 0.2–2.4%) / 2	–	–	–
**Hearing disorders**	1 (0.3%, 0.1–1.9%) / 1	1 (0.3%, 0.1–1.9%) / 1	–			
Ear congestion	1 (0.3%, 0.1–1.9%) / 1	1 (0.3%, 0.1–1.9%) / 1	–	–	–	–
**Disorders of respiratory system and mediastinal organs**	7 (2.3%, 1.1–4.8%) / 7	2 (0.7%, 0.2–2.4%) / 2	5 (1.7%, 0.7–3.9%) / 5	3 (3%, 1.0–8.5%) / 5	1 (1%, 0.2–5.4%) / 1	2 (2%, 0.6–7.0%) / 4
Throat pain	2 (0.7%, 0.2–2.4%) / 2	–	2 (0.7%, 0.2–2.4%) / 2	1 (1%, 0.2–5.4%) / 2	–	1 (1%, 0.2–5.4%) / 2
Pain in the oropharynx	1 (0.3%, 0.1–1.9%) / 1	–	1 (0.3%, 0.1–1.9%) / 1	1 (1%, 0.2–5.4%) / 1	–	1 (1%, 0.2–5.4%) / 1
Cough	1 (0.3%, 0.1–1.9%) / 1	–	1 (0.3%, 0.1–1.9%) / 1	1 (1%, 0.2–5.4%) / 1	–	1 (1%, 0.2–5.4%) / 1
Impaired sense of smell	1 (0.3%, 0.1–1.9%) / 1	1 (0.3%, 0.1–1.9%) / 1	–	–	–	–
Dyspnea	1 (0.3%, 0.1–1.9%) / 1	–	1 (0.3%, 0.1–1.9%) / 1	–	–	–
Sore throat	1 (0.3%, 0.1–1.9%) / 1	1 (0.3%, 0.1–1.9%) / 1	–	1 (1%, 0.2–5.4%) / 1	1 (1%, 0.2–5.4%) / 1	–
**Metabolic disorders**	3 (1%, 0.3–2.9%) / 9	–	3 (1%, 0.3–2.9%) / 9	1 (1%, 0.2–5.4%) / 1	–	1 (1%, 0.2–5.4%) / 1
Impaired appetite	3 (1%, 0.3–2.9%) / 9	–	3 (1%, 0.3–2.9%) / 9	1 (1%, 0.2–5.4%) / 1	–	1 (1%, 0.2–5.4%) / 1
**Other**	1 (0.3%, 0.1–1.9%) / 1	–	1 (0.3%, 0.1–1.9%) / 1			
Death from acute circulatory disorder	1 (0.3%, 0.1–1.9%) / 1	–	1 (0.3%, 0.1–1.9%) / 1			

Supplementary Table S3. Adverse events observed in the Vaccine Group and in the Placebo Group within 28 days following each vaccination by relation to vaccination

Adverse event	Relation to vaccination	Vaccine<br>Number of participants with AEs (%, 95% CI) / number of AEs			Placebo<br>Number of participants with AEs (%, 95% CI) / number of AEs		
		Total participants<br>N=298	Within 28 days after		Total participants<br>N=100	Within 28 days after	
			First dose	Second dose		First dose	Second dose
**Local and systemic reactions**	Definite	47 (15.8%, 12.1–20.3%) / 58	31 (10.4%, 7.4–14.4%) / 32	25 (8.4%, 5.7–12.1%) / 26	11 (11%, 6.3–18.6%) / 14	5 (5%, 2.2–11.2%) / 6	7 (7%, 3.4–13.7%) / 8
	Probable	3 (1%, 0.3–2.9%) / 3	1 (0.3%, 0.1–1.9%) / 1	2 (0.7%, 0.2–2.4%) / 2	1 (1%, 0.2–5.4%) / 4	–	1 (1%, 0.2–5.4%) / 4
	Possible	5 (1.7%, 0.7–3.9%) / 6	3 (1%, 0.3–2.9%) / 4	2 (0.7%, 0.2–2.4%) / 2			
	Unlikely	3 (1%, 0.3–2.9%) / 3		3 (1%, 0.3–2.9%) / 3	1 (1%, 0.2–5.4%) / 1		1 (1%, 0.2–5.4%) / 1
	Unrelated	5 (1.7%, 0.7–3.9%) / 13	2 (0.7%, 0.2–2.4%) / 2	3 (1%, 0.3–2.9%) / 11	2 (2%, 0.6–7.0%) / 2	2 (2%, 0.6–7.0%) / 2	
	Unknown	1 (0.3%, 0.1–1.9%) / 1	–	1 (0.3%, 0.1–1.9%) / 1	1 (1%, 0.2–5.4%) / 2	–	1 (1%, 0.2–5.4%) / 2
Pain at the injection site	Definite	46 (15.4%, 11.8–20.0%) / 54	30 (10.1%, 7.1–14.0%) / 30	24 (8.1%, 5.5–11.7%) / 24	10 (10%, 5.5–17.4%) / 11	5 (5%, 2.2–11.2%) / 5	6 (6%, 2.8–12.5%) / 6
	Probable				1 (1%, 0.2–5.4%) / 1	–	1 (1%, 0.2–5.4%) / 1
	Possible	2 (0.7%, 0.2–2.4%) / 2	1 (0.3%, 0.1–1.9%) / 1	1 (0.3%, 0.1–1.9%) / 1			
Induration at the injection site	Definite	3 (1%, 0.3–2.9%) / 3	1 (0.3%, 0.1–1.9%) / 1	2 (0.7%, 0.2–2.4%) / 2	2 (2%, 0.6–7.0%) / 2	–	2 (2%, 0.6–7.0%) / 2
	Probable				1 (1%, 0.2–5.4%) / 1		1 (1%, 0.2–5.4%) / 1
Hematoma at the injection site	Definite	–	–	–	1 (1%, 0.2–5.4%) / 1	1 (1%, 0.2–5.4%) / 1	–
Swelling at the injection site	Probable				1 (1%, 0.2–5.4%) / 1		1 (1%, 0.2–5.4%) / 1
Itching at the injection site	Probable				1 (1%, 0.2–5.4%) / 1		1 (1%, 0.2–5.4%) / 1
Fever	Definite	1 (0.3%, 0.1–1.9%) / 1	1 (0.3%, 0.1–1.9%) / 1	–	–	–	–
	Probable	3 (1%, 0.3–2.9%) / 3	1 (0.3%, 0.1–1.9%) / 1	2 (0.7%, 0.2–2.4%) / 2	–		–
	Possible	3 (1%, 0.3–2.9%) / 3	2 (0.7%, 0.2–2.4%) / 2	1 (0.3%, 0.1–1.9%) / 1	–	–	–
	Unlikely	2 (0.7%, 0.2–2.4%) / 2	–	2 (0.7%, 0.2–2.4%) / 2	–	–	–
	Unrelated	4 (1.3%, 0.5–3.4%) / 5	2 (0.7%, 0.2–2.4%) / 2	2 (0.7%, 0.2–2.4%) / 3	2 (2%, 0.6–7.0%) / 2	2 (2%, 0.6–7.0%) / 2	–
Malaise	Possible	1 (0.3%, 0.1–1.9%) / 1	1 (0.3%, 0.1–1.9%) / 1	–	–	–	–
	Unlikely	1 (0.3%, 0.1–1.9%) / 1		1 (0.3%, 0.1–1.9%) / 1	1 (1%, 0.2–5.4%) / 1		1 (1%, 0.2–5.4%) / 1
	Unrelated	2 (0.7%, 0.2–2.4%) / 8	–	2 (0.7%, 0.2–2.4%) / 8	1 (1%, 0.2–5.4%) / 2	–	1 (1%, 0.2–5.4%) / 2
	Unknown	1 (0.3%, 0.1–1.9%) / 1		1 (0.3%, 0.1–1.9%) / 1			
**Laboratory methods**	Possible	2 (0.7%, 0.2–2.4%) / 5		2 (0.7%, 0.2–2.4%) / 5			
	Unlikely	1 (0.3%, 0.1–1.9%) / 1	1 (0.3%, 0.1–1.9%) / 1	–			
	Unrelated	19 (6.4%, 4.1–9.7%) / 19	7 (2.3%, 1.1–4.8%) / 7	12 (4%, 2.3–6.9%) / 12	7 (7%, 3.4–13.7%) / 7	2 (2%, 0.6–7.0%) / 2	5 (5%, 2.2–11.2%) / 5
Increased leukocytes content	Unlikely	1 (0.3%, 0.1–1.9%) / 1	1 (0.3%, 0.1–1.9%) / 1	–	–	–	–
Positive SARS-CoV-2 PCR test	Unrelated	18 (6%, 3.9–9.3%) / 18	7 (2.3%, 1.1–4.8%) / 7	11 (3.7%, 2.1–6.5%) / 11	6 (6%, 2.8–12.5%) / 6	2 (2%, 0.6–7.0%) / 2	4 (4%, 1.6–9.8%) / 4
Increased creatine phosphokinase level	Possible	2 (0.7%, 0.2–2.4%) / 2		2 (0.7%, 0.2–2.4%) / 2			
	Unrelated	1 (0.3%, 0.1–1.9%) / 1	–	1 (0.3%, 0.1–1.9%) / 1	1 (1%, 0.2–5.4%) / 1	–	1 (1%, 0.2–5.4%) / 1
Increased ALT level	Possible	1 (0.3%, 0.1–1.9%) / 1	–	1 (0.3%, 0.1–1.9%) / 1	–	–	–
Increased AST level	Possible	1 (0.3%, 0.1–1.9%) / 1	–	1 (0.3%, 0.1–1.9%) / 1	–	–	–
Increased CRP level	Possible	1 (0.3%, 0.1–1.9%) / 1	–	1 (0.3%, 0.1–1.9%) / 1	–	–	–
**Blood and lymphatic system disorders**	Unlikely	1 (0.3%, 0.1–1.9%) / 1	1 (0.3%, 0.1–1.9%) / 1	–			
Inguinal lymphadenitis	Unlikely	1 (0.3%, 0.1–1.9%) / 1	1 (0.3%, 0.1–1.9%) / 1	–	–	–	–
**Blood vessel disorders**					1 (1%, 0.2–5.4%) / 1	1 (1%, 0.2–5.4%) / 1	–
Deep venous leg thrombosis	Unrelated	–	–	–	1 (1%, 0.2–5.4%) / 1	1 (1%, 0.2–5.4%) / 1	–
**Infections and parasitic invasions**	Possible	1 (0.3%, 0.1–1.9%) / 1	1 (0.3%, 0.1–1.9%) / 1	–			
	Unlikely	1 (0.3%, 0.1–1.9%) / 1	–	1 (0.3%, 0.1–1.9%) / 1			
	Unrelated	27 (9.1%, 6.3–12.9%) / 27	17 (5.7%, 3.6–8.9%) / 17	10 (3.4%, 1.8–6.1%) / 10	4 (4%, 1.6–9.8%) / 4	2 (2%, 0.6–7.0%) / 2	2 (2%, 0.6–7.0%) / 2
SARS-CoV-2 infection	Unrelated	25 (8.4%, 5.7–12.1%) / 25	16 (5.4%, 3.3–8.5%) / 16	9 (3%, 1.6–5.6%) / 9	4 (4%, 1.6–9.8%) / 4	2 (2%, 0.6–7.0%) / 2	2 (2%, 0.6–7.0%) / 2
Candidiasis	Unlikely	1 (0.3%, 0.1–1.9%) / 1	–	1 (0.3%, 0.1–1.9%) / 1	–	–	–
Other upper respiratory tract infections	Possible	1 (0.3%, 0.1–1.9%) / 1	1 (0.3%, 0.1–1.9%) / 1	–			
	Unrelated	2 (0.7%, 0.2–2.4%) / 2	1 (0.3%, 0.1–1.9%) / 1	1 (0.3%, 0.1–1.9%) / 1			
**Nervous system disorders**	Possible	6 (2%, 0.9–4.3%) / 7	3 (1%, 0.3–2.9%) / 3	4 (1.3%, 0.5–3.4%) / 4	1 (1%, 0.2–5.4%) / 1	1 (1%, 0.2–5.4%) / 1	–
	Unlikely	2 (0.7%, 0.2–2.4%) / 3	–	2 (0.7%, 0.2–2.4%) / 3	1 (1%, 0.2–5.4%) / 1	1 (1%, 0.2–5.4%) / 1	–
	Unrelated	4 (1.3%, 0.5–3.4%) / 9		4 (1.3%, 0.5–3.4%) / 9	3 (3%, 1.0–8.5%) / 8	1 (1%, 0.2–5.4%) / 1	2 (2%, 0.6–7.0%) / 7
	Unknown	1 (0.3%, 0.1–1.9%) / 1	1 (0.3%, 0.1–1.9%) / 1	–	1 (1%, 0.2–5.4%) / 2		1 (1%, 0.2–5.4%) / 2

Adverse event	Relation to vaccination	Vaccine<br>Number of participants with AEs (%, 95% CI) / number of AEs			Placebo<br>Number of participants with AEs (%, 95% CI) / number of AEs		
		Total participants<br>N=298	Within 28 days after		Total participants<br>N=100	Within 28 days after	
			First dose	Second dose		First dose	Second dose
Headache	Possible	6 (2%, 0.9–4.3%) / 6	3 (1%, 0.3–2.9%) / 3	3 (1%, 0.3–2.9%) / 3	1 (1%, 0.2–5.4%) / 1	1 (1%, 0.2–5.4%) / 1	–
	Unlikely	2 (0.7%, 0.2–2.4%) / 3	–	2 (0.7%, 0.2–2.4%) / 3	1 (1%, 0.2–5.4%) / 1	1 (1%, 0.2–5.4%) / 1	–
	Unrelated	4 (1.3%, 0.5–3.4%) / 9		4 (1.3%, 0.5–3.4%) / 9	3 (3%, 1.0–8.5%) / 8	1 (1%, 0.2–5.4%) / 1	2 (2%, 0.6–7.0%) / 7
	Unknown	1 (0.3%, 0.1–1.9%) / 1	1 (0.3%, 0.1–1.9%) / 1	–	1 (1%, 0.2–5.4%) / 2		1 (1%, 0.2–5.4%) / 2
Dizziness	Possible	1 (0.3%, 0.1–1.9%) / 1	–	1 (0.3%, 0.1–1.9%) / 1	–	–	–
**Musculoskeletal and connective tissue disorders**	Possible	2 (0.7%, 0.2–2.4%) / 4	1 (0.3%, 0.1–1.9%) / 2	1 (0.3%, 0.1–1.9%) / 2			
	Unlikely	1 (0.3%, 0.1–1.9%) / 2		1 (0.3%, 0.1–1.9%) / 2	1 (1%, 0.2–5.4%) / 1	1 (1%, 0.2–5.4%) / 1	–
	Unrelated	2 (0.7%, 0.2–2.4%) / 2	1 (0.3%, 0.1–1.9%) / 1	1 (0.3%, 0.1–1.9%) / 1			
Arthralgia	Possible	2 (0.7%, 0.2–2.4%) / 2	1 (0.3%, 0.1–1.9%) / 1	1 (0.3%, 0.1–1.9%) / 1	–	–	–
	Unlikely	1 (0.3%, 0.1–1.9%) / 1		1 (0.3%, 0.1–1.9%) / 1			
Lumbar pain	Unlikely	–	–	–	1 (1%, 0.2–5.4%) / 1	1 (1%, 0.2–5.4%) / 1	–
Myalgia	Possible	2 (0.7%, 0.2–2.4%) / 2	1 (0.3%, 0.1–1.9%) / 1	1 (0.3%, 0.1–1.9%) / 1	–	–	–
	Unlikely	1 (0.3%, 0.1–1.9%) / 1		1 (0.3%, 0.1–1.9%) / 1			
	Unrelated	2 (0.7%, 0.2–2.4%) / 2	1 (0.3%, 0.1–1.9%) / 1	1 (0.3%, 0.1–1.9%) / 1			
**Gastrointestinal tract disorders**	Possible	2 (0.7%, 0.2–2.4%) / 2		2 (0.7%, 0.2–2.4%) / 2			
	Unlikely	2 (0.7%, 0.2–2.4%) / 2	1 (0.3%, 0.1–1.9%) / 1	1 (0.3%, 0.1–1.9%) / 1			
	Unrelated				1 (1%, 0.2–5.4%) / 1	–	1 (1%, 0.2–5.4%) / 1
Diarrhea	Possible	1 (0.3%, 0.1–1.9%) / 1	–	1 (0.3%, 0.1–1.9%) / 1			
	Unrelated				1 (1%, 0.2–5.4%) / 1	–	1 (1%, 0.2–5.4%) / 1
Pyrosis	Unlikely	1 (0.3%, 0.1–1.9%) / 1	1 (0.3%, 0.1–1.9%) / 1	–	–	–	–
Nausea	Possible	1 (0.3%, 0.1–1.9%) / 1	–	1 (0.3%, 0.1–1.9%) / 1	–	–	–
	Unlikely	1 (0.3%, 0.1–1.9%) / 1	–	1 (0.3%, 0.1–1.9%) / 1	–	–	–
**Hearing disorders**	Unlikely	1 (0.3%, 0.1–1.9%) / 1	1 (0.3%, 0.1–1.9%) / 1	–			
Ear congestion	Unlikely	1 (0.3%, 0.1–1.9%) / 1	1 (0.3%, 0.1–1.9%) / 1	–	–	–	–
**Disorders of respiratory system and mediastinal organs**	Unlikely	2 (0.7%, 0.2–2.4%) / 2	1 (0.3%, 0.1–1.9%) / 1	1 (0.3%, 0.1–1.9%) / 1	1 (1%, 0.2–5.4%) / 1	1 (1%, 0.2–5.4%) / 1	–
	Unrelated	5 (1.7%, 0.7–3.9%) / 5	1 (0.3%, 0.1–1.9%) / 1	4 (1.3%, 0.5–3.4%) / 4	1 (1%, 0.2–5.4%) / 3	–	1 (1%, 0.2–5.4%) / 3
	Unknown				1 (1%, 0.2–5.4%) / 1	–	1 (1%, 0.2–5.4%) / 1
Throat pain	Unrelated	2 (0.7%, 0.2–2.4%) / 2	–	2 (0.7%, 0.2–2.4%) / 2	1 (1%, 0.2–5.4%) / 2	–	1 (1%, 0.2–5.4%) / 2
Pain in the oropharynx	Unrelated	1 (0.3%, 0.1–1.9%) / 1	–	1 (0.3%, 0.1–1.9%) / 1	–	–	–
	Unknown				1 (1%, 0.2–5.4%) / 1	–	1 (1%, 0.2–5.4%) / 1
Cough	Unlikely	1 (0.3%, 0.1–1.9%) / 1	–	1 (0.3%, 0.1–1.9%) / 1			
	Unknown				1 (1%, 0.2–5.4%) / 1	–	1 (1%, 0.2–5.4%) / 1
Impaired sense of smell	Unrelated	1 (0.3%, 0.1–1.9%) / 1	1 (0.3%, 0.1–1.9%) / 1	–	–	–	–
Dyspnea	Unrelated	1 (0.3%, 0.1–1.9%) / 1	–	1 (0.3%, 0.1–1.9%) / 1	–	–	–
Sore throat	Unlikely	1 (0.3%, 0.1–1.9%) / 1	1 (0.3%, 0.1–1.9%) / 1	–	1 (1%, 0.2–5.4%) / 1	1 (1%, 0.2–5.4%) / 1	–
**Metabolic disorders**	Possible	1 (0.3%, 0.1–1.9%) / 1		1 (0.3%, 0.1–1.9%) / 1			
	Unlikely	1 (0.3%, 0.1–1.9%) / 1		1 (0.3%, 0.1–1.9%) / 1			
	Unrelated	1 (0.3%, 0.1–1.9%) / 5	–	1 (0.3%, 0.1–1.9%) / 5	1 (1%, 0.2–5.4%) / 1	–	1 (1%, 0.2–5.4%) / 1
Impaired appetite	Possible	1 (0.3%, 0.1–1.9%) / 1		1 (0.3%, 0.1–1.9%) / 1			–
	Unlikely	1 (0.3%, 0.1–1.9%) / 1		1 (0.3%, 0.1–1.9%) / 1			
	Unrelated	1 (0.3%, 0.1–1.9%) / 5	–	1 (0.3%, 0.1–1.9%) / 5	1 (1%, 0.2–5.4%) / 1	–	1 (1%, 0.2–5.4%) / 1
**Other**	Unlikely	1 (0.3%, 0.1–1.9%) / 1	–	1 (0.3%, 0.1–1.9%) / 1			
Death from acute circulatory disorder	Unlikely	1 (0.3%, 0.1–1.9%) / 1	–	1 (0.3%, 0.1–1.9%) / 1			

Supplementary Table S4. Total adverse events observed in the 60+ age cohort within 21 days following each vaccination

Adverse event	Number of participants with AEs (%) / number of AEs			
	Total participants N=199	Within 21 days after		
		First dose	Second dose	Third dose
**Local and systemic reactions**	87 (43.5%) / 267	68 (34%) / 115	51 (25.5%) / 84	38 (19%) / 68
Asthenia	5 (2.5%) / 5	4 (2%) / 4	1 (0.5%) / 1	–
Pain	3 (1.5%) / 3	–	1 (0.5%) / 1	2 (1%) / 2
Pain at the injection site	74 (37%) / 123	56 (28%) / 56	40 (20.1%) / 43	24 (17%) / 24
Itching at the injection site	12 (6%) / 15	4 (2%) / 4	4 (2%) / 4	7 (3.5%) / 7
Chills	9 (4.5%) / 11	6 (3%) / 7	3 (1.5%) / 3	1 (0.5%) / 1
Swelling at the injection site	5 (2.5%) / 7	1 (0.5%) / 1	2 (1%) / 3	3 (1.5%) / 3
Foreign body sensation	1 (0.5%) / 1	1 (0.5%) / 1	–	–
Fever	8 (4%) / 8	4 (2%) / 4	1 (0.5%) / 1	3 (1.5%) / 3
Malaise	1 (0.5%) / 1	–	1 (0.5%) / 1	–
Induration at the injection site	12 (6%) / 18	4 (2%) / 4	7 (3.5%) / 8	6 (3%) / 6
Fatigue	34 (17%) / 64	25 (12.5%) / 30	14 (7%) / 17	14 (7%) / 17
Discomfort at the injection site	1 (0.5%) / 1	1 (0.5%) / 1	–	–
Erythema at the injection site	9 (4.5%) / 10	3 (1.5%) / 3	2 (1%) / 2	5 (2.5%) / 5
**Laboratory methods**	150 (75%) / 318	72 (36%) / 99	63 (31.5%) / 77	89 (44.5%) / 142
Proteinuria	6 (3%) / 6	3 (1.5%) / 3	–	3 (1.5%) / 3
Glycosuria	1 (0.5%) / 1	–	–	1 (0.5%) / 1
Leukocytes in urine	9 (4.5%) / 10	2 (1%) / 2	3 (1.5%) / 3	5 (2.5%) / 5
Abnormal ALT level	3 (1.5%) / 3	3 (1.5%) / 3	–	–
Abnormal glucose level	1 (0.5%) / 1	–	–	1 (0.5%) / 1
Abnormal urea level	3 (1.5%) / 3	–	1 (0.5%) / 1	2 (1%) / 2
Abnormal CRP level	6 (3%) / 7	1 (0.5%) / 1	1 (0.5%) / 1	4 (2%) / 5
Abnormal cholesterol level	3 (1.5%) / 3	–	–	3 (1.5%) / 3
Abnormal blood ALP level	5 (2.5%) / 6	–	3 (1.5%) / 3	2 (1%) / 3
Increased arterial blood pressure	6 (3%) / 11	4 (2%) / 5	3 (1.5%) / 3	2 (1%) / 3
Increased diastolic blood pressure	10 (5%) / 10	5 (2.5%) / 5	4 (2%) / 4	1 (0.5%) / 1
Increased relative density of urine	1 (0.5%) / 1	–	–	1 (0.5%) / 1
Increased systolic blood pressure	9 (4.5%) / 9	5 (2.5%) / 5	3 (1.5%) / 3	1 (0.5%) / 1
Increased erythrocyte sedimentation rate	4 (2%) / 4	2 (1%) / 2	1 (0.5%) / 1	1 (0.5%) / 1
Increased ALT level	11 (5.5%) / 12	4 (2%) / 4	1 (0.5%) / 1	6 (3%) / 7
Increased AST level	13 (6.5%) / 16	5 (2.5%) / 5	2 (1%) / 2	7 (3.5%) / 9
Increased bilirubin level	10 (5%) / 12	3 (1.5%) / 3	4 (2%) / 4	5 (2.5%) / 5
Increased glucose level	12 (6%) / 15	4 (2%) / 4	4 (2%) / 4	6 (3%) / 7
Increased creatinine level	3 (1.5%) / 3	1 (0.5%) / 1	1 (0.5%) / 1	1 (0.5%) / 1
Increased creatine phosphokinase level	18 (9%) / 19	5 (2.5%) / 5	6 (3%) / 6	8 (4%) / 8
Increased urea level	12 (6%) / 13	2 (1%) / 2	3 (1.5%) / 3	8 (4%) / 8
Increased CRP level	6 (3%) / 6	2 (1%) / 2	3 (1.5%) / 3	1 (0.5%) / 1

Adverse event	Number of participants with AEs (%) / number of AEs			
	Total participants N=199	Within 21 days after		
		First dose	Second dose	Third dose
Increased transaminase levels	1 (0.5%) / 1	–	1 (0.5%) / 1	–
Increased cholesterol level	31 (15.5%) / 36	4 (2%) / 4	12 (6%) / 12	19 (9.5%) / 20
Increased ALP level	5 (2.5%) / 6	2 (1%) / 2	1 (0.5%) / 1	3 (1.5%) / 3
Increased basophil count	1 (0.5%) / 2	1 (0.5%) / 1	–	1 (0.5%) / 1
Increased leukocyte count	8 (4%) / 8	7 (3.5%) / 7	1 (0.5%) / 1	–
Increased eosinophil count	4 (2%) / 4	1 (0.5%) / 1	2 (1%) / 2	1 (0.5%) / 1
SARS-CoV-2 infection	2 (1%) / 2	–	1 (0.5%) / 1	1 (0.5%) / 1
Decreased hemoglobin level	4 (2%) / 4	–	–	4 (2%) / 4
Decreased glucose level	4 (2%) / 5	–	–	4 (2%) / 5
Decreased creatine phosphokinase level	1 (0.5%) / 1	–	–	1 (0.5%) / 1
Decreased total protein level	2 (1%) / 2	–	–	2 (1%) / 2
Decreased lymphocyte count	3 (1.5%) / 3	2 (1%) / 2	1 (0.5%) / 1	
Decreased neutrophil count	10 (5%) / 11	8 (4%) / 8	1 (0.5%) / 1	2 (1%) / 2
Decreased platelet count	2 (1%) / 2	1 (0.5%) / 1	1 (0.5%) / 1	–
Increased lymphocyte count	2 (1%) / 2	2 (1%) / 2	–	–
Cylindruria	1 (0.5%) / 1	–	1 (0.5%) / 1	–
Erythrocytes in urine	53 (26.5%) / 57	19 (9.5%) / 19	12 (6%) / 12	26 (13%) / 26
**Gastrointestinal tract disorders**	17 (8.5%) / 20	13 (6.5%) / 13	3 (1.5%) / 3	3 (1.5%) / 4
Abdominal discomfort	1 (0.5%) / 1	1 (0.5%) / 1	–	–
Abdominal pain	1 (0.5%) / 1	1 (0.5%) / 1	–	–
Lip pain	1 (0.5%) / 1	1 (0.5%) / 1	–	–
Diarrhea	8 (4%) / 9	4 (2%) / 4	2 (1%) / 2	3 (1.5%) / 3
Dyspepsia	2 (1%) / 2	2 (1%) / 2	–	–
Toothache	1 (0.5%) / 1	–	1 (0.5%) / 1	–
Nausea	5 (2.5%) / 5	4 (2%) / 4	–	1 (0.5%) / 1
**Infections and parasitic invasions**	31 (15.5%) / 31	9 (4.5%) / 9	9 (4.5%) / 9	13 (6.5%) / 13
Upper respiratory tract infections	10 (5%) / 10	1 (0.5%) / 1	6 (3%) / 6	3 (1.5%) / 3
SARS-CoV-2 infection	18 (9%) / 18	6 (3%) / 6	3 (1.5%) / 3	9 (4.5%) / 9
Nasal herpes simplex lesions	1 (0.5%) / 1	1 (0.5%) / 1	–	–
Herpes zoster	1 (0.5%) / 1	–	–	1 (0.5%) / 1
Labial herpes simplex lesions	1 (0.5%) / 1	1 (0.5%) / 1	–	–
**Metabolic and nutrition disorders**	16 (8%) / 20	6 (3%) / 6	2 (1%) / 2	11 (5.5%) / 12
Hyperglycemia	2 (1%) / 2	1 (0.5%) / 1	1 (0.5%) / 1	–
Hypoglycemia	11 (5.5%) / 11	1 (0.5%) / 1	1 (0.5%) / 1	9 (4.5%) / 9
Hypoproteinemia	7 (2.5%) / 7	4 (2%) / 4	–	3 (1.5%) / 3
**Disorders of respiratory system and mediastinal organs**	21 (10.5%) / 33	8 (4%) / 9	9 (4.5%) / 13	8 (4%) / 11
Pain in the oropharynx	5 (2.5%) / 5	1 (0.5%) / 1	2 (1%) / 2	2 (1%) / 2
Nasal congestion	4 (2%) / 4	1 (0.5%) / 1	2 (1%) / 2	1 (0.5%) / 1

Adverse event	Number of participants with AEs (%) / number of AEs			
	Total participants N=199	Within 21 days after		
		First dose	Second dose	Third dose
Cough	6 (3%) / 6	3 (1.5%) / 3	1 (0.5%) / 1	2 (1%) / 2
Dyspnea	1 (0.5%) / 1	–	1 (0.5%) / 1	–
Swelling of the pharynx	2 (1%) / 2	–	–	2 (1%) / 2
Sore throat	4 (2%) / 4	2 (1%) / 2	1 (0.5%) / 1	1 (0.5%) / 1
Rhinorrhea	10 (5%) / 10	2 (1%) / 2	5 (2.5%) / 5	3 (1.5%) / 3
Sneezing	1 (0.5%) / 1	–	1 (0.5%) / 1	–
**Skin and subcutaneous tissue disorders**	4 (2%) / 4	3 (1.5%) / 3	1 (0.5%) / 1	–
Hyperhidrosis	1 (0.5%) / 1	1 (0.5%) / 1	–	–
Itching	2 (1%) / 2	2 (1%) / 2	–	–
Rash	1 (0.5%) / 1	–	1 (0.5%) / 1	–
**Muscular. skeletal and connective tissue disorders**	27 (13.5%) / 38	17 (8.5%) / 19	8 (4%) / 9	9 (4. 5%) / 10
Arthralgia	5 (2.5%) / 6	2 (1%) / 2	2 (1%) / 2	1 (0.5%) / 2
Pain in the limb	2 (1%) / 2	1 (0.5%) / 1	–	1 (0.5%) / 1
Back pain	2 (1%) / 2	1 (0.5%) / 1	–	1 (0.5%) / 1
Myalgia	23 (11.5%) / 27	14 (7%) / 14	7 (3.5%) / 7	6 (3%) / 6
Musculoskeletal discomfort	1 (0.5%) / 1	1 (0.5%) / 1	–	–
**Nervous system disorders**	40 (20%) / 66	21 (10.5%) / 27	19 (9.5 %) / 23	11 (11%) / 16
Insomnia	3 (1.5%) / 3	1 (0.5%) / 1	2 (1%) / 2	–
Headache	35 (17.5%) / 54	16 (8%) / 21	16 (8%) / 19	10 (5%) / 14
Dizziness	4 (2%) / 4	2 (1%) / 2	1 (0.5%) / 1	1 (0.5%) / 1
Discomfort in the head	1 (0.5%) / 1	–	1 (0.5%) / 1	–
Syncope	1 (0.5%) / 1	–	–	1 (0.5%) / 1
Somnolence	2 (1%) / 3	2 (1%) / 3	–	–
**Eye disorders**	1 (0.5%) / 2	1 (0.5%) / 1	**–**	1 (0.5%) / 1
Hypersecretory lacrimation	1 (0.5%) / 1	1 (0.5%) / 1	–	–
Photopsy	1 (0.5%) / 1	–	–	1 (0.5%) / 1
**Hearing disorders**	2 (1%) / 2	1 (0.5%) / 1	–	1 (0.5%) / 1
Ear congestion	1 (0.5%) / 1	1 (0.5%) / 1	–	–
tinnitus	1 (0.5%) / 1	–	–	1 (0.5%) / 1
**Renal and urinary tract disorders**	11 (5.5%) / 11	7 (3.5%) / 7	1 (0.5%) / 1	3 (1.5%) / 3
Hematuria	2 (1%) / 2	1 (0.5%) / 1	1 (0.5%) / 1	–
Glycosuria	1 (0.5%) / 1	1 (0.5%) / 1	–	–
Leukocytes in urine	4 (2%) / 4	4 (2%) / 4	–	–
Proteinuria	4 (2%) / 4	1 (0.5%) / 1	–	3 (1.5%) / 3
**Heart disorders**	8 (4%) / 10	6 (3%) / 6	–	3 (1.5%) / 4
Arrhythmia	1 (0.5%) / 2	1 (0.5%) / 1	–	1 (0.5%) / 1
Chest pain	1 (0.5%) / 1	1 (0.5%) / 1	–	–

Adverse event	Number of participants with AEs (%) / number of AEs			
	Total participants N=199	Within 21 days after		
		First dose	Second dose	Third dose
Bradycardia	2 (1%) / 2	2 (1%) / 2	–	–
Discomfort in the heart	1 (0.5%) / 2	–	–	1 (0.5%) / 2
Stenocardia	2 (1%) / 2	1 (0.5%) / 1	–	1 (0.5%) / 1
Tachycardia	1 (0.5%) / 1	1 (0.5%) / 1	–	–
**Vascular disorders**	3 (1.5%) / 3	1 (0.5%) / 1	–	2 (1%) / 2
Hypertensive crisis	1 (0.5%) / 1	1 (0.5%) / 1	–	–
Blood vessel rupture	1 (0.5%) / 1	–	–	1 (0.5%) / 1
Nosebleed	1 (0.5%) / 1	–	–	1 (0.5%) / 1
**Injuries. intoxications and complications of procedures**	2 (1%) / 2	1 (0.5%) / 1	1 (0.5%) / 1	–
Fracture of the upper limb	1 (0.5%) / 1	1 (0.5%) / 1	–	–
Meniscus injury	1 (0.5%) / 1	–	1 (0.5%) / 1	–

Supplementary Table S5. Adverse events observed in the 60+ age cohort within 21 days following each vaccination by relation to vaccination

Adverse event	Relation to vaccination	Total participants N=199	Number of participants with AEs (%) / number of AEs		
			Within 21 days after		
			First dose	Second dose	Third dose
**Local and systemic reactions**	Definite	60 (30%) / 134	45 (22.5%) / 54	34 (17%) / 47	23 (11.5%) / 33
	Probable	23 (11.5%) / 38	13 (6.5%) / 15	11 (5.5%) / 13	8 (4%) / 10
	Possible	35 (17.5%) / 67	25 (12.5%) / 36	12 (6%) / 15	13 (6.5%) / 16
	Unlikely	8 (4%) / 8	4 (2%) / 4	2 (1%) / 2	2 (1%) / 2
	Unrelated	12 (6%) / 20	6 (3%) / 6	7 (3.5%) / 7	6 (3%) / 7
Asthenia	Possible	2 (1%) / 2	2 (1%) / 2	–	–
	Unlikely	1 (0.5%) / 1	1 (0.5%) / 1	–	–
	Unrelated	2 (1%) / 2	1 (0.5%) / 1	1 (0.5%) / 1	–
Pain	Possible	2 (1%) / 2	–	1 (0.5%) / 1	1 (0.5%) / 1
	Unrelated	1 (0.5%) / 1	–	–	1 (0.5%) / 1
Pain at the injection site	Definite	59 (29.5%) / 95	44 (22%) / 44	31 (15.6%) / 33	18 (9%) / 18
	Probable	18 (9%) / 26	11 (5.5%) / 11	9 (4.5%) / 9	6 (3%) / 6
	Possible	2 (1%) / 2	1 (0.5%) / 1	1 (0.5 %) /1	–
Itching at the injection site	Definite	8 (4%) / 10	2 (1%) / 2	4 (2%) / 4	4 (2%) / 4
	Probable	2 (1%) / 3	1 (0.5%) / 1	–	2 (1%) / 2
	Possible	2 (1%) / 2	1 (0.5%) / 1	–	1 (0.5%) / 1
Chills	Probable	1 (0.5%) / 1	1 (0.5%) / 1	–	–
	Possible	8 (4%) / 10	5 (2.5%) / 6	3 (1.5%) / 3	1 (0.5%) / 1
Swelling at the injection site	Definite	5 (2.5%) / 6	1 (0.5%) / 1	2 (1%) / 2	3 (1.5%) / 3
	Probable	1 (0.5%) / 1	–	1 (0.5%) / 1	–
Foreign body sensation	Unlikely	1 (0.5%) / 1	1 (0.5%) / 1	–	–
Fever	Probable	1 (0.5%) / 1	–	1 (0.5%) / 1	–
	Possible	4 (2%) / 4	3 (1.5%) / 3	–	1 (0.5%) / 1
	Unlikely	1 (0.5%) / 1	1 (0.5%) / 1	–	–
	Unrelated	2 (1%) / 2	–	–	2 (1%) / 2
Malaise	Unrelated	1 (0.5%) / 1	–	1 (0.5%) / 1	–
Induration at the injection site	Definite	12 (6%) / 16	4 (2%) / 4	6 (3%) / 7	5 (2.5%) / 5
	Probable	2 (1%) / 2	–	1 (0.5%) / 1	1 (0.5%) / 1
Fatigue	Probable	2 (1%) / 3	2 (1%) / 2	1 (0.5%) / 1	–
	Possible	27 (13.5%) / 44	19 (9.5%) / 22	9 (3.5%) / 10	11 (5.5%) / 12
	Unlikely	4 (2%) / 4	1 (0.5%) / 1	2 (1%) / 2	1 (0.5%) / 1
	Unrelated	9 (4.5%) / 13	5 (2.5%) / 5	4 (2%) / 4	4 (2%) / 4
Discomfort at the injection site	Possible	1 (0.5%) / 1	1 (0.5%) / 1	–	–
Erythema at the injection site	Definite	7 (3.5%) / 7	3 (1.5%) / 3	1 (0.5%) / 1	3 (1.5%) / 3
	Probable	3 (1.5%) / 3	–	1 (0.5%) / 1	2 (1%) / 2
**Laboratory methods**	Possible	22 (11%) / 28	9 (4.5%) / 12	9 (4.5%) / 10	6 (3%) / 6
	Unlikely	72 (36%) / 152	34 (17%) / 41	28 (14%) / 39	48 (24%) / 72

Adverse event	Relation to vaccination	Total participants N=199	Number of participants with AEs (%) / number of AEs		
			Within 21 days after		
			First dose	Second dose	Third dose
	Unrelated	78 (39%) / 138	36 (18%) / 46	27 (13.5%) / 28	42 (21%) / 64
Proteinuria	Unrelated	6 (3%) / 6	3 (1.5%) / 3	–	3 (1.5%) / 3
Glycosuria	Unrelated	1 (0.5%) / 1	–	–	1 (0.5%) / 1
Leukocytes in urine	Unlikely	8 (4%) / 9	1 (0.5%) / 1	3 (1.5%) / 3	5 (2.5%) / 5
	Unrelated	1 (0.5%) / 1	1 (0.5%) / 1	–	–
Abnormal ALT level	Unrelated	3 (1.5%) / 3	3 (1.5%) / 3	–	–
Abnormal glucose level	Unrelated	1 (0.5%) / 1	–	–	1 (0.5%) / 1
Abnormal urea level	Unrelated	3 (1.5%) / 3	–	1 (0.5%) / 1	2 (1%) / 2
Abnormal CRP level	Unrelated	6 (3%) / 7	1 (0.5%) / 1	1 (0.5%) / 1	4 (2%) / 5
Abnormal cholesterol level	Unrelated	3 (1.5%) / 3	–	–	3 (1.5%) / 3
Abnormal blood ALP level	Unrelated	5 (2.5%) / 6	–	3 (1.5%) / 3	2 (1%) / 3
Increased arterial blood pressure	Possible	1 (0.5%) / 1	–	–	1 (0.5%) / 1
	Unlikely	4 (2%) / 6	4 (2%) / 4	1 (0.5%) / 1	1 (0.5%) / 1
	Unrelated	3 (1.5%) / 4	1 (0.5%) / 1	2 (1%) / 2	1 (0.5%) / 1
Increased diastolic blood pressure	Unlikely	7 (3.5%) / 7	4 (2%) / 4	3 (1.5%) / 3	–
	Unrelated	3 (1.5%) / 3	1 (0.5%) / 1	1 (0.5%) / 1	1 (0.5%) / 1
Increased relative density of urine	Unlikely	1 (0.5%) / 1	–	–	1 (0.5%) / 1
Increased systolic blood pressure	Unlikely	2 (1%) / 2	1 (0.5%) / 1	1 (0.5%) / 1	–
	Unrelated	7 (3.5%) / 7	4 (2%) / 4	2 (1%) / 2	1 (0.5%) / 1
Increased erythrocyte sedimentation rate	Possible	1 (0.5%) / 1	–	1 (0.5%) / 1	–
	Unlikely	2 (1%) / 2	1 (0.5%) / 1	–	1 (0.5%) / 1
	Unrelated	1 (0.5%) / 1	1 (0.5%) / 1	–	–
Increased ALT level	Unlikely	8 (4%) / 9	4 (2%) / 4	–	4 (2%) / 5
	Unrelated	3 (1.5%) / 3	–	1 (0.5%) / 1	2 (1%) / 2
Increased AST level	Unlikely	10 (5%) / 12	4 (2%) / 4	2 (1%) / 2	5 (2.5%) / 6
	Unrelated	4 (2%) / 4	1 (0.5%) / 1	–	3 (1.5%) / 3
Increased bilirubin level	Unlikely	6 (3%) / 6	2 (1%) / 2	2 (1%) / 2	2 (1%) / 2
	Unrelated	5 (2.5%) / 6	1 (0.5%) / 1	2 (1%) / 2	3 (1.5%) / 3
Increased glucose level	Unlikely	9 (4.5%) / 11	3 (1.5%) / 3	2 (1%) / 2	5 (2.5%) / 6
	Unrelated	3 (1.5%) / 4	1 (0.5%) / 1	2 (1%) / 2	1 (0.5%) / 1
Increased creatinine level	Unlikely	1 (0.5%) / 1	–	1 (0.5%) / 1	–
	Unrelated	2 (1%) / 2	1 (0.5%) / 1	–	1 (0.5%) / 1
Increased creatine phosphokinase level	Possible	4 (2%) / 4	1 (0.5%) / 1	2 (1%) / 2	1 (0.5%) / 1
	Unlikely	11 (5.5%) / 11	1 (0.5%) / 1	4 (2%) / 4	6 (3%) / 6
	Unrelated	4 (2%) / 4	3 (1.5%) / 3	–	1 (0.5%) / 1
Increased urea level	Unlikely	8 (4%) / 9	2 (1%) / 2	2 (1%) / 2	5 (2.5%) / 5
	Unrelated	4 (2%) / 4	–	1 (0.5%) / 1	3 (1.5%) / 3
Increased CRP level	Possible	4 (2%) / 4	2 (1%) / 2	2 (1%) / 2	–

Adverse event	Relation to vaccination	Total participants N=199	Number of participants with AEs (%) / number of AEs		
			Within 21 days after		
			First dose	Second dose	Third dose
	Unlikely	2 (1%) / 2	–	1 (0.5%) / 1	1 (0.5%) / 1
Increased transaminase levels	Unrelated	1 (0.5%) / 1	–	1 (0.5%) / 1	–
Increased cholesterol level	Possible	1 (0.5%) / 1	1 (0.5%) / 1	–	–
	Unlikely	16 (8%) / 17	1 (0.5%) / 1	7 (3.5%) / 7	9 (4.5%) / 9
	Unrelated	14 (7%) / 18	2 (1%) / 2	5 (2.5%) / 5	10 (5%) / 11
Increased ALP level	Unlikely	3 (1.5%) / 4	1 (0.5%) / 1	1 (0.5%) / 1	2 (1%) / 2
	Unrelated	2 (1%) / 2	1 (0.5%) / 1	–	1 (0.5%) / 1
Increased basophil count	Possible	1 (0.5%) / 1	1 (0.5%) / 1	–	–
	Unrelated	1 (0.5%) / 1	–	–	1 (0.5%) / 1
Increased leukocyte count	Unrelated	8 (4%) / 8	7 (3.5%) / 7	1 (0.5%) / 1	–
Increased eosinophil count	Possible	3 (1.5%) / 3	1 (0.5%) / 1	2 (1%) / 2	–
	Unlikely	1 (0.5%) / 1	–	–	1 (0.5%) / 1
SARS-CoV-2 infection	Unrelated	2 (1%) / 2	–	1 (0.5%) / 1	1 (0.5%) / 1
Decreased hemoglobin level	Unlikely	2 (1%) / 2	–	–	2 (1%) / 2
	Unrelated	2 (1%) / 2	–	–	2 (1%) / 2
Decreased glucose level	Unrelated	4 (2%) / 5	–	–	4 (2%) / 5
Decreased creatine phosphokinase level	Unrelated	1 (0.5%) / 1	–	–	1 (0.5%) / 1
Decreased total protein level	Unlikely	1 (0.5%) / 1	–	–	1 (0.5%) / 1
	Unrelated	1 (0.5%) / 1	–	–	1 (0.5%) / 1
Decreased lymphocyte count	Unlikely	3 (1.5%) / 3	2 (1%) / 2	1 (0.5%) / 1	–
Decreased neutrophil count	Possible	4 (2%) / 4	3 (1.5%) / 3	1 (0.5%) / 1	–
	Unlikely	3 (1.5%) / 3	1 (0.5%) / 1	–	2 (1%) / 2
	Unrelated	4 (2%) / 4	4 (2%) / 4	–	–
Decreased platelet count	Unlikely	1 (0.5%) / 1	1 (0.5%) / 1	–	–
	Unrelated	1 (0.5%) / 1	–	1 (0.5%) / 1	–
Increased lymphocyte count	Possible	1 (0.5%) / 1	1 (0.5%) / 1	–	–
	Unrelated	1 (0.5%) / 1	1 (0.5%) / 1	–	–
Cylindruria	Unlikely	1 (0.5%) / 1	–	1 (0.5%) / 1	–
Erythrocytes in urine	Possible	7 (3.5%) / 8	2 (1%) / 2	2 (1%) / 2	4 (2%) / 4
	Unlikely	30 (15%) / 31	8 (4%) / 8	7 (3.5%) / 7	16 (8%) / 16
	Unrelated	16 (8%) / 18	9 (4.5%) / 9	3 (1.5%) / 3	6 (3%) / 6
**Gastrointestinal tract disorders**	Possible	5 (2.5%) / 6	4 (2%) / 4	–	1 (0.5%) / 2
	Unlikely	6 (3%) / 6	3 (1.5%) / 3	2 (1%) / 2	1 (0.5%) / 1
	Unrelated	8 (4%) / 8	6 (3%) / 6	1 (0.5%) / 1	1 (0.5%) / 1
Abdominal discomfort	Unrelated	1 (0.5%) / 1	1 (0.5%) / 1	–	–
Abdominal pain	Unlikely	1 (0.5%) / 1	1 (0.5%) / 1	–	–
Lip pain	Possible	1 (0.5%) / 1	1 (0.5%) / 1	–	–
Diarrhea	Possible	2 (1%) / 2	1 (0.5%) / 1	–	1 (0.5%) / 1

Adverse event	Relation to vaccination	Total participants N=199	Number of participants with AEs (%) / number of AEs		
			Within 21 days after		
			First dose	Second dose	Third dose
	Unlikely	2 (1%) / 2	–	1 (0.5%) / 1	1 (0.5%) / 1
	Unrelated	5 (2.5%) / 5	3 (1.5%) / 3	1 (0.5%) / 1	1 (0.5%) / 1
Dyspepsia	Unlikely	1 (0.5%) / 1	1 (0.5%) / 1	–	–
	Unrelated	1 (0.5%) / 1	1 (0.5%) / 1	–	–
Toothache	Unlikely	1 (0.5%) / 1	–	1 (0.5%) / 1	–
Nausea	Possible	3 (1.5%) / 3	2 (1%) / 2	–	1 (0.5%) / 1
	Unlikely	1 (0.5%) / 1	1 (0.5%) / 1	–	–
	Unrelated	1 (0.5%) / 1	1 (0.5%) / 1	–	–
**Infections and parasitic invasions**	Possible	1 (0.5%) / 1	1 (0.5%) / 1	–	–
	Unlikely	5 (2.5%) / 5	3 (1.5%) / 3	–	2 (1%) / 2
	Unrelated	25 (17.5%) / 25	5 (2.5%) / 5	9 (4.5%) / 9	11 (5.5%) / 11
Upper respiratory tract infections	Unlikely	2 (1%) / 2	1 (0.5%) / 1	–	1 (0.5%) / 1
	Unrelated	8 (4%) / 8	–	6 (3%) / 6	2 (1%) / 2
SARS-CoV-2 infection	Unlikely	2 (1%) / 2	1 (0.5%) / 1	–	1 (0.5%) / 1
	Unrelated	16 (8%) / 16	5 (2.5%) / 5	3 (1.5%) / 3	8 (4%) / 8
Nasal herpes simplex lesions	Unlikely	1 (0.5%) / 1	1 (0.5%) / 1	–	–
Herpes zoster	Unrelated	1 (0.5%) / 1	–	–	1 (0.5%) / 1
Labial herpes simplex lesions	Possible	1 (0.5%) / 1	1 (0.5%) / 1	–	–
**Metabolic and nutrition disorders**	Unlikely	11 (5.5%) / 13	4 (2%) / 4	–	8 (4%) / 9
	Unrelated	7 (3.5%) / 7	2 (1%) / 2	2 (1%) / 2	3 (1.5%) / 3
Hyperglycemia	Unlikely	1 (0.5%) / 1	1 (0.5%) / 1	–	–
	Unrelated	1 (0.5%) / 1	–	1 (0.5%) / 1	–
Hypoglycemia	Unlikely	7 (3.5%) / 7	1 (0.5%) / 1	–	6 (6%) / 6
	Unrelated	4 (2%) / 4	–	1 (0.5%) / 1	3 (1.5%) / 3
Hypoproteinemia	Unlikely	4 (2%) / 5	2 (1%) / 2	–	3 (1.5%) / 3
	Unrelated	2 (1%) / 2	2 (1%) / 2	–	–
**Disorders of respiratory system and mediastinal organs**	Possible	3 (1.5%) / 5	–	2 (1%) / 3	1 (0.5%) / 2
	Unlikely	9 (4.5%) / 12	4 (2%) / 5	3 (1.5%) / 5	2 (1%) / 2
	Unrelated	11 (5.5%) / 16	4 (2%) / 4	4 (2%) / 5	5 (2.5%) / 7
Pain in the oropharynx	Unlikely	2 (1%) / 2	1 (0.5%) / 1	1 (0.5%) / 1	–
	Unrelated	3 (1.5%) / 3	–	1 (0.5%) / 1	2 (1%) / 2
Nasal congestion	Possible	2 (1%) / 2	–	1 (0.5%) / 1	1 (0.5%) / 1
	Unrelated	2 (1%) / 2	1 (0.5%) / 1	1 (0.5%) / 1	–
Cough	Unlikely	4 (2%) / 4	2 (1%) / 2	1 (0.5%) / 1	1 (0.5%) / 1
	Unrelated	2 (1%) / 2	1 (0.5%) / 1	–	1 (0.5%) / 1
Dyspnea	Unlikely	1 (0.5%) / 1	–	1 (0.5%) / 1	–
Swelling of the pharynx	Unlikely	1 (0.5%) / 1	–	–	1 (0.5%) / 1
	Unrelated	1 (0.5%) / 1	–	–	1 (0.5%) / 1

Adverse event	Relation to vaccination	Total participants N=199	Number of participants with AEs (%) / number of AEs		
			Within 21 days after		
			First dose	Second dose	Third dose
Sore throat	Possible	1 (0.5%) / 1	–	–	1 (0.5%) / 1
	Unlikely	1 (0.5%) / 1	1 (0.5%) / 1	–	–
	Unrelated	2 (1%) / 2	1 (0.5%) / 1	1 (0.5%) / 1	–
Rhinorrhea	Possible	1 (0.5%) / 1	–	1 (0.5%) / 1	–
	Unlikely	2 (1%) / 2	1 (0.5%) / 1	2 (1%) / 2	–
	Unrelated	6 (6%) / 6	1 (0.5%) / 1	2 (1%) / 2	3 (1.5%) / 3
Sneezing	Possible	1 (0.5%) / 1	–	1 (0.5%) / 1	–
**Skin and subcutaneous tissue disorders**	Possible	2 (1%) / 2	1 (0.5%) / 1	1 (0.5%) / 1	–
	Unlikely	1 (0.5%) / 1	1 (0.5%) / 1	–	–
	Unrelated	1 (0.5%) / 1	1 (0.5%) / 1	–	–
Hyperhidrosis	Unlikely	1 (0.5%) / 1	1 (0.5%) / 1	–	–
Itching	Possible	1 (0.5%) / 1	1 (0.5%) / 1	–	–
	Unrelated	1 (0.5%) / 1	1 (0.5%) / 1	–	–
Rash	Possible	1 (0.5%) / 1	–	1 (0.5%) / 1	–
**Muscular. skeletal and connective tissue disorders**	Probable	4 (2%) / 4	1 (0.5%) / 1	3 (1.5%) / 3	–
	Possible	15 (7.5%) / 17	8 (4%) / 9	3 (1.5%) / 3	5 (2.5%) / 5
	Unlikely	3 (1.5%) / 3	2 (1%) / 2	–	1 (0.5%) / 1
	Unrelated	9 (4.5%) / 14	6 (3%) / 7	2 (1%) / 3	3 (1.5%) / 4
Arthralgia	Possible	1 (0.5%) / 1	–	1 (0.5%) / 1	–
	Unrelated	4 (2%) / 5	2 (1%) / 2	1 (0.5%) / 1	1 (0.5%) / 2
Pain in the limb	Unlikely	1 (0.5%) / 1	–	–	1 (0.5%) / 1
	Unrelated	1 (0.5%) / 1	1 (0.5%) / 1	–	–
Back pain	Possible	1 (0.5%) / 1	1 (0.5%) / 1	–	–
	Unrelated	1 (0.5%) / 1	–	–	1 (0.5%) / 1
Myalgia	Probable	3 (1.5%) / 4	1 (0.5%) / 1	3 (1.5%) / 3	–
	Possible	15 (7.5%) / 15	8 (4%) / 8	2 (1%) / 2	5 (2.5%) / 5
	Unlikely	2 (1%) / 2	2 (1%) / 2	–	–
	Unrelated	5 (2.5%) / 6	3 (1.5%) / 3	2 (1%) / 2	1 (0.5%) / 1
Musculoskeletal discomfort	Unrelated	1 (0.5%) / 1	1 (0.5%) / 1	–	–
**Nervous system disorders**	Probable	3 (1.5%) / 3	1 (0.5%) / 1	2 (1%) / 2	–
	Possible	22 (11%) / 29	10 (5%) / 11	9 (4.5%) / 9	8 (4%) / 9
	Unlikely	10 (5%) / 11	5 (2.5%) / 5	4 (2%) / 4	2 (1%) / 2
	Unrelated	13 (6.5%) / 23	7 (3.5%) / 10	6 (3%) / 8	3 (1.5%) / 5
Insomnia	Unlikely	3 (1.5%) / 3	1 (0.5%) / 1	2 (1%) / 2	–
Headache	Probable	3 (1.5%) / 3	1 (0.5%) / 1	2 (1%) / 2	–
	Possible	21 (10.5%) / 28	9 (4.5%) / 10	9 (4.5%) / 9	8 (4%) / 9
	Unlikely	4 (2%) / 4	2 (1%) / 2	2 (1%) / 2	–
	Unrelated	12 (6%) / 19	5 (2.5%) / 8	5 (2.5%) / 6	3 (1.5%) / 5

Adverse event	Relation to vaccination	Total participants N=199	Number of participants with AEs (%) / number of AEs		
			Within 21 days after		
			First dose	Second dose	Third dose
Dizziness	Unlikely	3 (1.5%) / 3	2 (1%) / 2	–	1 (0.5%) / 1
	Unrelated	1 (0.5%) / 1	–	1 (0.5%) / 1	–
Discomfort in the head	Unrelated	1 (0.5%) / 1	–	1 (0.5%) / 1	–
Syncope	Unlikely	1 (0.5%) / 1	–	–	1 (0.5%) / 1
Somnolence	Possible	1 (0.5%) / 1	1 (0.5%) / 1	–	–
	Unrelated	2 (1%) / 2	2 (1%) / 2	–	–
**Eye disorders**	Unrelated	1 (0.5%) / 2	1 (0.5%) / 1	–	1 (0.5%) / 1
Hypersecretory lacrimation	Unrelated	1 (0.5%) / 1	1 (0.5%) / 1	–	–
Photopsy	Unrelated	1 (0.5%) / 1	–	–	1 (0.5%) / 1
**Hearing disorders**	Possible	1 (0.5%) / 1	–	–	1 (0.5%) / 1
	Unrelated	1 (0.5%) / 1	1 (0.5%) / 1	–	–
Ear congestion	Unrelated	1 (0.5%) / 1	1 (0.5%) / 1	–	–
tinnitus	Possible	1 (0.5%) / 1	–	–	1 (0.5%) / 1
**Renal and urinary tract disorders**	Possible	2 (1%) / 2	2 (1%) / 2	–	–
	Unlikely	7 (3.5%) / 7	4 (2%) / 4	–	3 (1.5%) / 3
	Unrelated	2 (1%) / 2	1 (0.5%) / 1	1 (0.5%) / 1	–
Hematuria	Unlikely	1 (0.5%) / 1	1 (0.5%) / 1	–	–
	Unrelated	1 (0.5%) / 1	–	1 (0.5%) / 1	–
Glycosuria	Unrelated	1 (0.5%) / 1	1 (0.5%) / 1	–	–
Leukocytes in urine	Unlikely	4 (2%) / 4	4 (2%) / 4	–	–
Proteinuria	Unlikely	4 (2%) / 4	1 (0.5%) / 1	–	3 (1.5%) / 3
**Heart disorders**	Unlikely	3 (1.5%) / 4	3 (1.5%) / 3	–	1 (0.5%) / 1
	Unrelated	5 (2.5%) / 6	3 (1.5%) / 3	–	2 (1%) / 3
Arrhythmia	Unlikely	1 (0.5%) / 2	1 (0.5%) / 1	–	1 (0.5%) / 1
Chest pain	Unlikely	1 (0.5%) / 1	1 (0.5%) / 1	–	–
Bradycardia	Unrelated	2 (1%) / 2	2 (1%) / 2	–	–
Discomfort in the heart	Unrelated	1 (0.5%) / 2	–	–	1 (0.5%) / 2
Stenocardia	Unlikely	1 (0.5%) / 1	1 (0.5%) / 1	–	–
	Unrelated	1 (0.5%) / 1	–	–	1 (0.5%) / 1
Tachycardia	Unrelated	1 (0.5%) / 1	1 (0.5%) / 1	–	–
**Vascular disorders**	Unrelated	3 (1.5%) / 3	1 (0.5%) / 1	–	2 (1%) / 2
Hypertensive crisis	Unrelated	1 (0.5%) / 1	1 (0.5%) / 1	–	–
Blood vessel rupture	Unrelated	1 (0.5%) / 1	–	–	1 (0.5%) / 1
Nosebleed	Unrelated	1 (0.5%) / 1	–	–	1 (0.5%) / 1
**Injuries. intoxications and complications of procedures**	Unlikely	1 (0.5%) / 1	–	1 (0.5%) / 1	–
	Unrelated	1 (0.5%) / 1	1 (0.5%) / 1	–	–
Fracture of the upper limb	Unrelated	1 (0.5%) / 1	1 (0.5%) / 1	–	–
Meniscus injury	Unlikely	1 (0.5%) / 1		1 (0.5%) / 1	–

Supplementary Table S6. Proportion of participants of the 18–60 age cohort with detectable anti-SARS-CoV-2 antibodies in NT and CMIA at different time points after the first vaccination

Group	N	Test	Positive, n (%, 95% CI)					
			Screening	Day 7	Day 14	Day 21 (7 days after the 2nd vaccination)	Day 28 (14 days after the 2nd vaccination)	Day 42 (28 days after the 2nd vaccination)
Vaccine	122	nAB (NT)	53 (43.4, 35.0–52.3)	68 (55.7, 46.9–64.2)	92 (75.4, 67.1–82.2)	112 (91.8, 85.6–95.5)	112 (91.8, 85.6–95.5)	113 (92.6, 86.6–96.1)
	101	anti-RBD IgG (Beckman)	28 (28, 20.1–37.5)[1]	33 (32.7, 24.3–42.3)	43 (42.6, 33.4–52.3)	44 (44.9, 35.4–54.8)[2]	47 (46.5, 37.1–56.2)	44 (43.6, 34.3–53.3)
	101	anti-N IgG (Abbot)	24 (24, 16.7–33.2)[1]	28 (27.7, 19.9–37.1)	34 (33.7, 25.2–43.3)	43 (43.9, 34.5–53.7)[2]	57 (56.4, 46.7–65.7)	56 (55.4, 45.7–64.8)
Placebo	45	nAB (NT)	17 (37.8, 25.1–52.4)	18 (40.0, 27.0–54.6)	19 (42.2, 29.0–56.7)	17 (38.6, 25.7–53.4)[3]	17 (37.8, 25.1–52.4)	18 (40.9, 27.7–55.6)[3]
	36	anti-RBD IgG (Beckman)	10 (27.8, 15.8–44)	11 (30.6, 18–46.9)	11 (30.6, 18–46.9)	11 (30.6, 18–46.9)	11 (30.6, 18–46.9)	9 (25, 13.8–41.1)
	36	anti-N IgG (Abbot)	10 (27.8, 15.8–44)	10 (27.8, 15.8–44)	8 (22.2, 11.7–38.1)	9 (25, 13.8–41.1)	8 (22.2, 11.7–38.1)	8 (22.2, 11.7–38.1)

[1] N = 100; [2] N = 98; [3] N = 44

Due to the lack of defined correlates of protection against COVID-19, initially we determined the optimal method of anti-SARS-CoV-2 humoral immunogenicity assessment by comparing the neutralizing antibody (nAB) detection rates in NT with two commercial CMIA tests commonly used for the screening of anti-N protein IgG (Architect SARS-CoV-2 IgG, Abbott Diagnostics, USA) and anti-RBD IgG (Access SARS-CoV-2 IgG, Beckman, USA). We used NT and both CMIA tests to analyze serum samples from all 167 participants of the 18–60 age cohort (122 from the Vaccine Group and 45 from the Placebo Group) included in Stage 3, obtained at screening and at Days 7, 14, 21, 28 and 42 after the 1st immunization (Supplementary Table S6). The analysis included both the participants who were anti-SARS-CoV-2 nAB negative (69/122 in Vaccine Group and 28/45 in Placebo Group) and anti-SARS-CoV-2 nAB positive (53/122 in Vaccine Group and 17/45 in Placebo Group) at screening.

There was no strong correlation between seropositivity rates assessed in NT and CMIA at all time points. Generally, the serum sample with the higher nAB titers produced higher signal in CMIA tests. However, several NT positive serum samples were negative in CMIA with SARS-CoV-2 RBD.

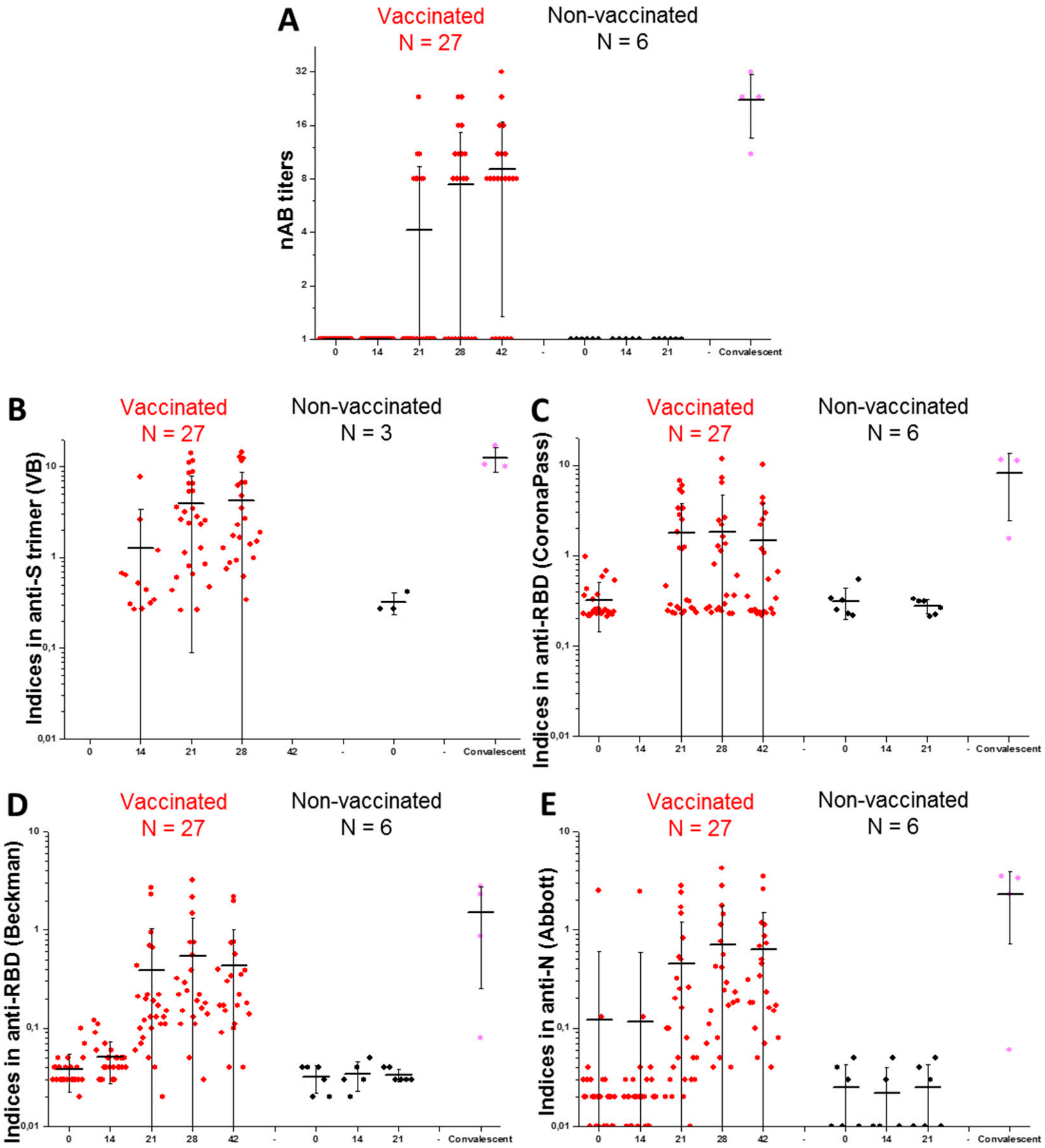

Supplementary Figure S1. Anti-SARS-CoV-2 antibody levels following immunization of 27 randomly picked participants who were seronegative at screening. nAB titers in NT (A); and positivity coefficients for ELISA and CMIA kits: anti-S trimer total antibodies ELISA (Vector-Best) (B); anti-RBD total antibodies ELISA (CoronaPass) (C); anti-RBD IgG CMIA (Beckman) (D); and anti-N IgG CMIA (Abbot) (E). Black line – Mean, whiskers – SD. Blue area signifies a positive threshold (grey zone) for each test. Black dots represent the results from sera samples from 6 randomly picked participants from Placebo Group obtained in the same tests in parallel (with Vector-Best sera from 3 participants were tested). Purple dots represent 4 samples of convalescent sera used as positive controls.

In order to assess the sensitivity of commercial CMIA and ELISA tests, serum samples from 27 randomly picked participants from the Vaccine Group in Stage 3 of the 18–60 cohort who were anti-SARS-CoV-2 nAB negative at screening were used to evaluate the correlation between the virus neutralization titers in NT and the positivity coefficients

in binding antibody detection assays. Two abovementioned CMIA tests and two ELISA-based tests detecting total antibodies to S trimer expressed in eukaryotic system and total antibodies to RBD (SARS-CoV-2-AB total-EIA-BEST, Vector-Best, Russia and CoronaPass Total kit, Genetico, Russia, respectively) were used (Supplementary Figure S1).

Both ELISA-based total antibody detection kits showed higher correlation of seropositivity rates and positivity coefficients with the virus neutralization titers in NT than the CMIA tests. The Vector-Best ELISA kit was able to detect total antibodies to S trimer in 3/27 participants at Day 14 after the 1st immunization (Supplementary Figure S1B), while virus-neutralizing antibodies in NT (Supplementary Figure S1A) and total antibodies to RBD (Supplementary Figure S1C) were detected only from Day 21.

The analysis of the individual test results showed that in the samples with detectable nAB levels (over 1:8) we could see a significant increase in the positivity coefficients in CMIA kits compared with the samples obtained at screening (Supplementary Figure S1A,D,E), but the levels were below the “grey zone”, indicating insufficient sensitivity of these tests.
